# Meta-research: How many diagnostic or prognostic models published in radiological journals are evaluated externally?

**DOI:** 10.1007/s00330-023-10168-3

**Published:** 2023-09-12

**Authors:** Maira Hameed, Jason Yeung, Darren Boone, Sue Mallett, Steve Halligan

**Affiliations:** Centre for Medical Imaging, University College London UCL, Charles Bell House, 43-45 Foley Street, London, W1W 7TS UK

**Keywords:** Prognosis, Models statistical, Proportional hazards models, Logistic models, Evaluation study

## Abstract

**Objectives:**

Prognostic and diagnostic models must work in their intended clinical setting, proven via “external evaluation”, preferably by authors uninvolved with model development. By systematic review, we determined the proportion of models published in high-impact radiological journals that are evaluated subsequently.

**Methods:**

We hand-searched three radiological journals for multivariable diagnostic/prognostic models 2013–2015 inclusive, developed using regression. We assessed completeness of data presentation to allow subsequent external evaluation. We then searched literature to August 2022 to identify external evaluations of these index models.

**Results:**

We identified 98 index studies (73 prognostic; 25 diagnostic) describing 145 models. Only 15 (15%) index studies presented an evaluation (two external). No model was updated. Only 20 (20%) studies presented a model equation. Just 7 (15%) studies developing Cox models presented a risk table, and just 4 (9%) presented the baseline hazard. Two (4%) studies developing non-Cox models presented the intercept. Just 20 (20%) articles presented a Kaplan–Meier curve of the final model. The 98 index studies attracted 4224 citations (including 559 self-citations), median 28 per study. We identified just six (6%) subsequent external evaluations of an index model, five of which were external evaluations by researchers uninvolved with model development, and from a different institution.

**Conclusions:**

Very few prognostic or diagnostic models published in radiological literature are evaluated externally, suggesting wasted research effort and resources. Authors’ published models should present data sufficient to allow external evaluation by others. To achieve clinical utility, researchers should concentrate on model evaluation and updating rather than continual redevelopment.

**Clinical relevance statement:**

The large majority of prognostic and diagnostic models published in high-impact radiological journals are never evaluated. It would be more efficient for researchers to evaluate existing models rather than practice continual redevelopment.

**Key Points:**

• *Systematic review of highly cited radiological literature identified few diagnostic or prognostic models that were evaluated subsequently by researchers uninvolved with the original model.*

• *Published radiological models frequently omit important information necessary for others to perform an external evaluation: Only 20% of studies presented a model equation or nomogram.*

• *A large proportion of research citing published models focuses on redevelopment and ignores evaluation and updating, which would be a more efficient use of research resources.*

**Graphical abstract:**

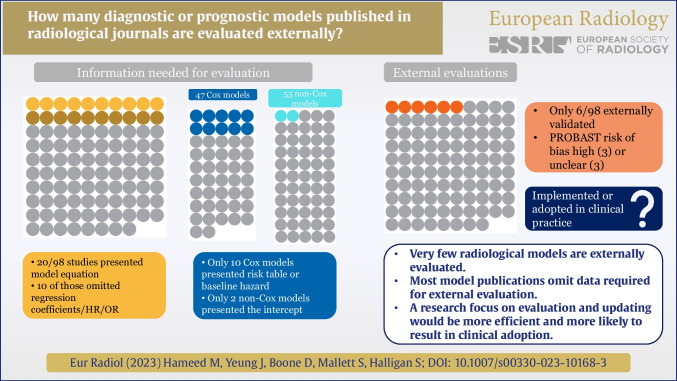

**Supplementary information:**

The online version contains supplementary material available at 10.1007/s00330-023-10168-3.

## Introduction

The medical literature is experiencing a tsunami of diagnostic and prognostic models. Radiological journals are bursting with models that claim clinical utility, for example, the ability of MR imaging to predict subsequent outcomes [1–3]. A recent narrative “Viewpoint” noted “exponential” model publication, blaming easy dataset availability combined with inexpensive computational power, and stated most were clinically useless because researchers lacked methodological expertise to develop and evaluate models properly [4]. Specifically, poor development encourages bias that risks overfitting, culminating in models inaccurate for new patients [5].

To be useful, models must work in their intended clinical setting. The pivotal step towards this is “external evaluation” (“external validation”), whereby model performance is evaluated in representative patients not used for development. This contrasts with “internal evaluation”, where development data is reused for evaluation. It is unlikely that clinicians will adopt models unless proven accurate in patients similar to their own. Despite this, model research emphasises development and ignores evaluation [6]. Most models go unused because they have never been evaluated externally, or fail this test [7]. It follows that useful models will have passed external evaluation. External evaluation is especially relevant to radiomic models, where biomarkers must be consistent across institutions [8]. To eliminate “allegiance bias”, evaluation is best performed by researchers who did not develop the model [9], which requires the published model to report enough data to allow this [10].

Research effort is wasted if published models are never used, but the extent to which this applies to radiological journals is unknown. Our primary aim was to determine by systematic review how often models underwent external evaluation by others. Secondary aims were to identify whether models presented an internal evaluation and/or information sufficient to allow external evaluation by others.

## Materials and methods

Ethical permission is not required by our institution for systematic review of primary literature. Our research is reported using PRISMA guidelines [11] (Supplementary Material).

### Eligibility

We hypothesised that if an index model publication omitted external evaluation, researchers or clinicians wishing to use the model would perform their own evaluation subsequently. If so, it is likely some such evaluations would be published and reference the index model. We therefore identified published models and searched subsequent literature for external evaluations. Eligible index publications described diagnostic or prognostic multivariable models in humans, incorporating imaging biomarkers (with/without non-imaging biomarkers) and claiming potential clinical utility (all disciplines). We restricted our search to models developed using regression techniques, and did not aim to investigate machine-learning methods.

### Information sources

Since our primary interest was radiological models, we searched the top three indexed (Scopus) general radiology journals publishing original research (*Radiology*, *Investigative Radiology*, *European Radiology*) hypothesising that models published here would be more methodologically sound than those of lesser journals. This procedure also reduced the volume of data, rendering the search feasible. We searched The Web of Science and The National Library of Science via PubMed.

### Search

We hypothesised that 50 index models would provide representative data. We identified index models published in print 2013 to 2015 inclusive then searched until August 2022 for subsequent external evaluations. M.H. hand searched all journal contents pages, while J.Y. and D.B. searched half each, independently.

### Study selection

We identified titles using the following the terms: “prognostic”, “prognosis”, “prognostically”, “predictive”, “prediction”, “predicts”, “predicting”, “predictor”, “predictors”, “predictable”, “model”, “models”, “modelling”, “external validation”, and “external clinical validation” and then applied eligibility criteria to the abstract.

### Data collection

The following data were extracted from index models (refined after a pilot of 10): diagnostic or predictive; model type (linear/logistic/Cox); clinical application; outcomes; number of patients/events; and total factors assessed (imaging/other). We extracted information necessary for external evaluation, e.g. regression coefficients/hazard ratios; model equation and/or Kaplan–Meier curve; risk tables for Cox models, and terms “prognostic index” and “baseline hazard” [10]. We noted if the index model included evaluation and, if so, the type. Using “cited by” in Web of Science (Clarivate), we then identified all publications citing the index model, noting self-citations. Via the abstract, we determined whether the subsequent publication described external evaluation of the index model, retrieving the full text if so, or where there was uncertainty. External evaluation was defined by including the factors and weightings used by the index model (prior to any updating), in different patients, from a different source. Evaluation methodology and any updating/additional factors were extracted. We noted if authors were unrelated to the index model. Uncertainty was resolved by face-to-face discussion**.**

### Risk of bias

Risk-of-bias assessment for external evaluations used PROBAST [12].

### Summary measures and synthesis

We performed descriptive analysis summarising review findings as median, interquartile range (IQR), and range.

## Results

### Model characteristics

**Fig. 1:**
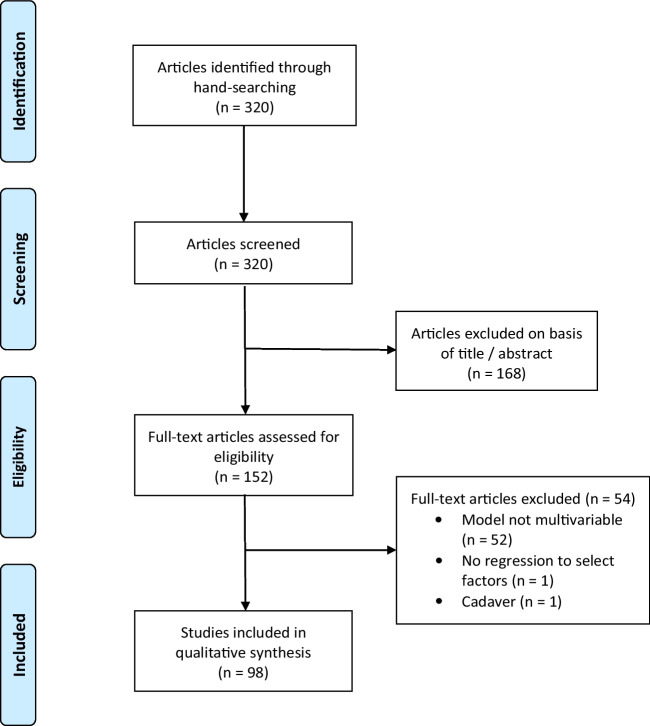
PRISMA diagram of article selection for the systematic review [11]

We identified 320 articles describing potential models (Fig. 1). Of 152 full texts assessed, 54 were excluded (52 non-multivariable; 1 non-regression; 1 non-human), leaving 98 index publications (twice our a priori target; Electronic Supplementary Material 1). Publication frequency increased with time: 2013, 26; 2014, 33; and 2015, 39. Seventy-three (74%) studies were prognostic and 25 (26%), diagnostic. Gastrointestinal (including hepato-biliary/pancreatic) was the most studied system (21, 21%), followed by thorax (13, 13%), with cardiovascular/neurological joint third (11, 11%). Malignancy was the commonest topic, 64 (65%) of all studies.

The 98 studies described 145 individual models; 71 (72%) described one model, 20 (20%) described two models, 3 (3%) described three models, and 4 (4%) studies described 4, 5, 6, and 10 models respectively. Forty-three (44%) studies developed Cox models, 39 (40%) developed logistic, 11 (11%) developed linear, 1 (1%) developed Poisson, and 4 (4%) developed both Cox and logistic models. Multiple outcomes were modelled: The three commonest were overall survival (37, 38%), disease-free survival (14, 14%), and cardiovascular events (12, 12%). MRI variables were modelled in 44 (45%) studies, CT in 40 (41%), PET/PET-CT in 10 (10%), and ultrasound in just 3 (3%). Eight (8%) studies modelled data from multiple imaging modalities. The median number of patients per study was 98, range 19 [13] to 11,462 [14]. Most (85, 87%) studies modelled data per patient. Thirteen (13%) modelled per lesion (or per eye [15], artery [16], procedure [17]).

**Fig. 2:**
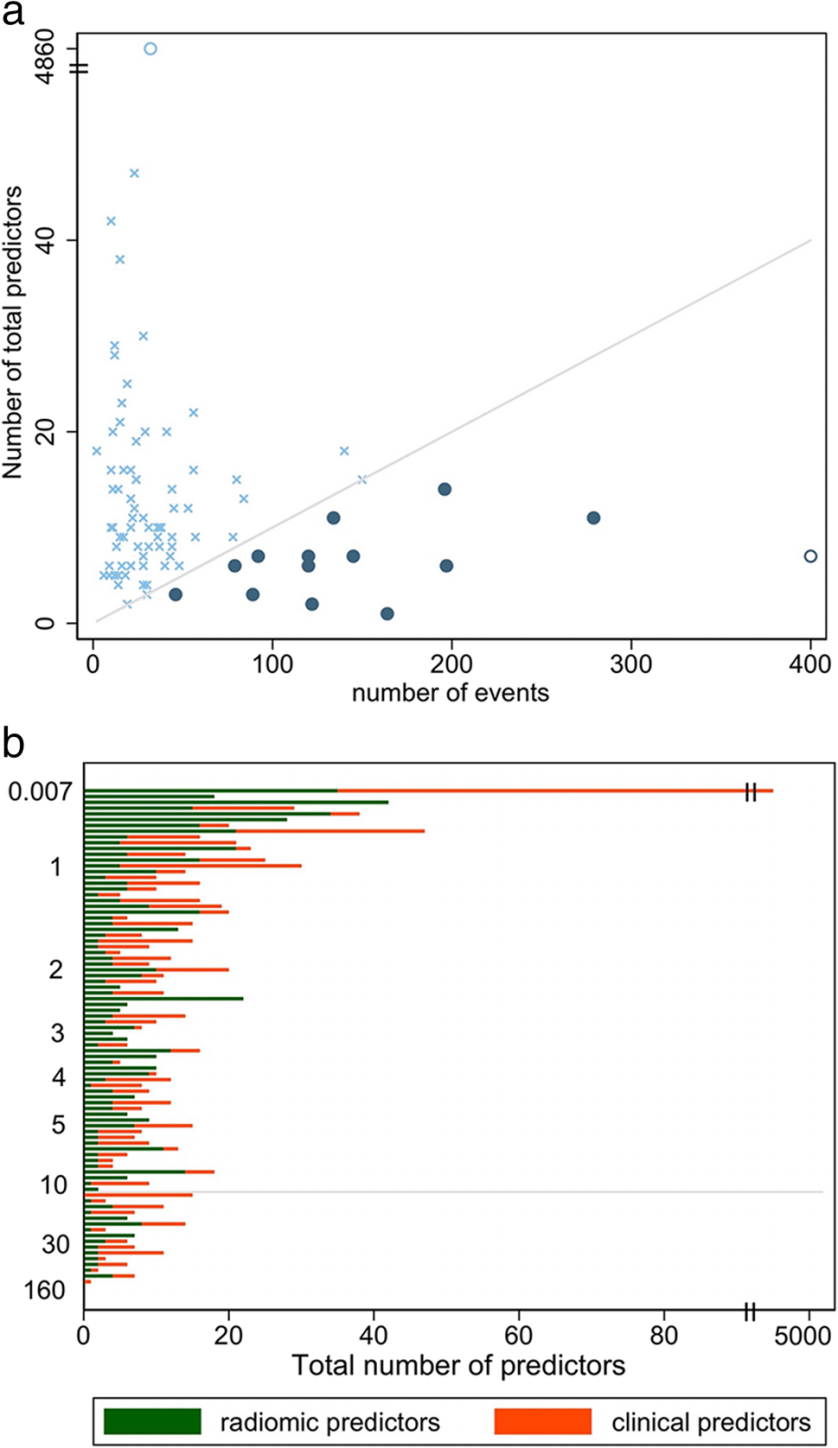
**a** Scatterplot of studies included in the systematic review. The *x*-axis indicates the number of patient events per study, and the *y*-axis indicates the total number of predictor variables per study. Studies above the threshold (crosses) appear underpowered whereas those below (dots) appear adequately powered. **b** Bar chart of individual research articles where the *x*-axis illustrates the number of individual radiomic/imaging variables and clinical variables per study. The *y*-axis describes the number of variables that should be studied according to the “rule of thumb”, which requires at least 10 patient events per variable. Studies above the horizontal line appear underpowered

For 50 of 55 (91%) studies employing non-Cox models, we could estimate the number of events (i.e. numerically smallest outcome group). The median was 28 events (IQR 18 to 56, range 2 [18] to 279 [19]). The median number of imaging variables investigated was 6 (IQR 2 to 9, range 0 [20] to 42 [21]); the study without imaging variables investigated clinical variables to predict CT outcomes [20]. The median number of non-imaging variables was 2.5, (IQR 1 to 8, range 0 to 26 [21]). Indeed, 18 (36%) studies excluded non-imaging variables. Overall, the total number of variables per study was median 9, (IQR 6 to 16, range 1 [22] to 47 [17]). Using the “rule of ten” [23], only 9 (18%) studies appeared adequately powered [19, 20, 22, 24–29] (Fig. 2). Details of variables non-significant in univariate analysis were omitted by 24 (24%) studies.

### Author evaluation

Only 15 (15%) studies presented an evaluation alongside index models: 7 used internal cross-evaluation [15, 30–35], 4 used temporal evaluation [17, 20, 36, 37], 2 combined internal and temporal [38, 39], and only 2 used external evaluation [40, 41]. No model was updated.

### External evaluation

Regarding data necessary to permit external evaluation, 20 (20%) studies presented the model equation (or nomogram) in print or online [13, 15–17, 26, 29, 36–39, 41–50]. Regression coefficients/hazard ratios/odds ratios for individual variables ultimately included in the model were omitted by 10 (50%) articles [44, 51–59]. Of the 47 studies describing Cox models, only 7 (15%) presented the risk table [4, 14, 37, 54, 60–62] and just 6 (13%) presented the baseline hazard [33, 41, 43, 63–65]. Of the 55 studies describing non-Cox models, only 2 (4%) presented the intercept (necessary to evaluate absolute risk probabilities) in the text [17, 26]. While the term “prognostic index” appeared in just 2 (2%) articles [66, 67], neither specified the value for their own model. A Kaplan–Meier curve of the final model was presented in 20 (20%) articles.

The 98 studies attracted 4224 citations, median 28 per study (IQR 19 to 49, range 3 [68] to 270 [69]). Forty-five were non-English. Five hundred fifty-nine (13%) were self-citations. Only five [17, 20, 35, 37, 41] of the 98 study models were subsequently externally evaluated (one evaluated twice [35]), i.e. six (6%) external evaluations [70–75]. Three did not state “validation” or “evaluation” in the title or abstract [71, 72, 75]. A radiogenomic model of renal cancer [37] was evaluated subsequently by the same authors, using patients recruited prospectively from a different institution [71]. The remaining five evaluations did not include authors from the original publication. Two of these developed their own model alongside the evaluation: A model to predict head CT features from clinical factors [20] was evaluated externally on 5296 cases [70], alongside redevelopment of a second model. A nomogram to predict survival following selective internal radiation therapy [41] was evaluated before development of a new model for thermal ablation [72]. A model predicting complications following renal cryoablation [17] was externally evaluated in 201 patients from another institution [73]. A CT model of blunt abdominal trauma [35] was evaluated by two groups, one of whom found it superior to other scores [74], and another who supplemented it using repeated CT [75].

### PROBAST assessment

**Fig. 3:**
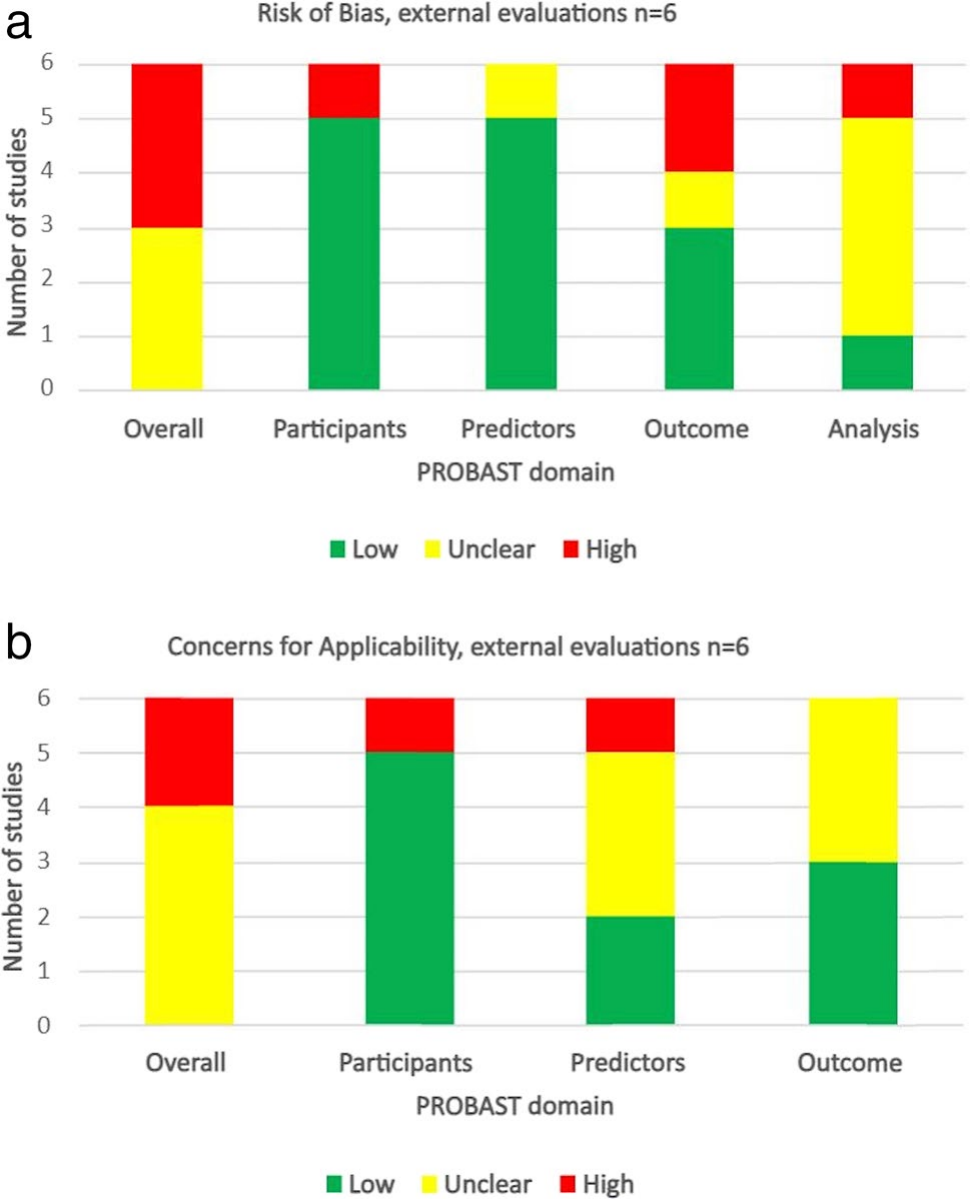
PROBAST assessment [12] of the 6 external evaluations for (**a**) risk of bias and (**b**) concerns for applicability. Studies are categorised into either low, unclear, or high for each domain (participants, predictors, outcomes, analysis, and overall)

PROBAST [12] assessment of all six external evaluations for risk of bias is shown in Fig. 3a. Overall risk of bias was “high” for three evaluations and “unclear” for the remaining three. None attracted a “low” risk of bias. Applicability scores are shown in Fig. 3b. Similarly, no external evaluation attracted a “low” overall score for applicability concerns: Two evaluations were considered “high risk” and the remaining four “unclear”.

## Discussion

Various methods assess model performance. Internal evaluation reuses data already exploited for development while temporal evaluation uses patients from the same source, but recruited at different times; both overestimate performance. External evaluation uses patients from different centres, potentially different clinical pathways, and even different countries (“geographic” evaluation), and avoids “allegiance bias” if researchers are uninvolved with the original development [9]. Fundamentally, external evaluation replicates research results to ensure they are “true”, a cornerstone of the scientific method [10]. We avoid the term “validation”, which implies success. “Evaluation” is preferable, resulting in a “valid” or “invalid” model, depending on outcome [5, 10].

Multivariable models presently comprise a substantial proportion of imaging research, and publication is accelerating as access to data processing increases: The number of models published annually increased during our search period. Our primary objective was to determine if research effort is wasted because these models go unused. Ultimately, we found that the large majority of published models are never evaluated: From 98 index articles, we identified just six external evaluations following model publication, five of which originated from researchers unrelated to model development (two evaluated the same model). This suggests most models never enter clinical practice since publications demonstrating “real world” utility are mandatory for implementation [9]. We found that only 15% of index model publications incorporated an evaluation of any description (and most were internal and/or temporal), stressing the need for subsequent external evaluation. Our findings also suggest that authors do not perform later external evaluations of their own models. Obtaining external data may be a disincentive and authors may lack methodological skills or motivation, especially if they believe new models will be easier to publish. One model [38] prompted a Letter-To-The-Editor asking why there was no evaluation [76]. The authors agreed that implementation required “additional experience with its use in a large cohorts of patients” but expressed no intention to do this [76].

The dearth of external evaluations also suggest that index models lack scientific credibility, do not answer a useful clinical question, or report insufficient data to permit evaluation. Regarding scientific credibility, like others [77], we found model development usually underpowered, with most investigating excessive factors versus patient events. A typical example examined 47 predictors, but with just 23 events in 56 patients, the authors were powered to investigate only two [47]. TRIPOD (Transparent Reporting of a multivariable prediction model for Individual Prognosis Or Diagnosis) is explicit that authors, “Present the full prediction model to allow predictions for individuals (i.e. all regression coefficients, and model intercept or baseline survival at a given time point)” [78]. The model equation is simply the mathematical combination of variables and their weightings. Publishing a model without the equation is akin to publishing a recipe without the quantity of ingredients. Models should present regression coefficient/odds ratio for all variables, and the intercept [79]. Cox models estimate survival relative to baseline survival, so survival at given time-points requires the cumulative baseline hazard [10, 78]. It is also desirable to present Kaplan-Meier curves for the groups predicted by the model (as opposed to for individual predictors) [10]. Nevertheless, we found most studies presented insufficient data for others to attempt evaluation. Only 20% presented an equation/nomogram, and even fewer explained its interpretation. Just 13% of Cox models reported the baseline hazard. Although “prognostic index” is the main product of a Cox model [10], we identified this term in just two articles, and then only in their discussion. Non-significant univariate analyses were often omitted, something not recommended, especially with low event rates [80]. Omission means that readers cannot determine the complete range of factors investigated nor assess power or overfitting risks. Omission also frustrates systematic review of investigated factors. Furthermore, using a two-step approach to exclude variables based on univariate analysis is considered statistically flawed [80].

External evaluation determines “discrimination” for new patients, namely how accurately events are separated overall, for example disease/no-disease? “Calibration” describes accuracy for individual diagnoses/predictions. Discrimination is more important because models can be re-calibrated. Fundamentally, external evaluation does not entail re-development using new data. Rather, evaluation employs the same factors and weightings used by the index model. The model may then be updated in the light of the evaluation, by re-weighting established factors, or adding new factors [81]. We identified only two external evaluations that attempted recalibration [70, 72]. Statisticians argue it is more efficient to update existing models [6], but this advice is usually ignored; over 60 different models predict breast cancer outcomes [82]. We found that most workers citing published models invariably repeated development, creating new models “from scratch” using new univariate analyses. This simply creates yet another unevaluated model and does not advance the field. For example, a model predicting axillary lymph node metastases from breast cancer via PET/CT [19] was not evaluated, with subsequent researchers choosing instead to redevelop a new model to answer the same question [83]. Rather than evaluate their own model predicting lung cancer [65], the same authors subsequently redeveloped another model with additional variables [84]. Instead, it would be more efficient to evaluate the first model and then determine if prediction improved when new variables are added. Failure to evaluate existing models is regrettable because combining older development data with new information increases model stability.

As a secondary aim, we assessed identified external evaluations for risk of bias and applicability concerns using PROBAST [12]. We found all six attracted “high” or “unclear” risk for both these domains, suggesting that evaluations themselves are methodologically questionable. For example, evaluation of a model developed to predict survival following selective internal radiation therapy for liver metastases [41] was evaluated in patients treated with thermal ablation, which appears illogical [72]. An index model to predict surgical intervention following blunt abdominal trauma [35] was evaluated in patients in whom “significant injury” was undefined [74]. Via systematic review, Collins found that most external evaluations were poorly designed and reported themselves [85].

Our review does have limitations. We investigated models developed by radiologists, published in imaging journals, ignoring imaging models in non-radiological journals. However, as radiologists ourselves, we were interested in the fate of models published in our journals. We concentrated on highly cited journals, hypothesising these were most likely to report high-quality models deserving external evaluation. Searching all radiological journals would be prohibitively intensive for little additional return. We wished simply to accrue a representative sample of models sufficient to answer our hypothesis (doubling our a priori target of 50). While we initially intended to concentrate on prognostic models, around one-quarter of models identified were diagnostic, and we included these; prediction and diagnosis should not be confounded. We allowed a generous time horizon following model publication so as to capture all subsequent evaluations: While it is possible index model reporting quality improved subsequent to 2015, advancement would need to be dramatic to alter our findings. Authors might argue that not all multivariable models claim clinical utility, with some simply “predictor finding”. If so, we would question the point of predictor finding if clinical utility is not the eventual aim. For example, radiomic factors result in numerical values with no meaning for individualised prognosis/diagnosis unless portrayed in an understandable format, e.g. within a multivariable model. Models are also required to combine multiple factors in an interpretable fashion. Ultimately, our review suggests that predictor finding is not translating to individualised patient care, although we accept that failure to identify a published external evaluation does not prove that the model was never used clinically. We are also aware that some researchers consider “traditional” regression-based models inferior to those developed using machine learning, claiming the latter are more accurate, and a search period subsequent to ours would undoubtedly identify a greater proportion of such models. We excluded just one model because development did not use regression. Also, systematic review suggests that machine-learning models are neither more accurate [86] nor reported more comprehensively than regression-based models [87].

In summary, systematic review suggests that very few prognostic or diagnostic models published in the radiological literature are evaluated externally, either by the original researchers or by others. This may arise because authors present insufficient detail to permit evaluation by others, because models are not scientifically credible or do not answer a useful clinical question, or because evaluation is perceived as arduous, unproductive, or less likely to culminate in scientific publication. Authors should report models with sufficient methods to allow external evaluation, via adherence to TRIPOD guidelines (78). Ultimately, to best use a scarce research resource, it would be more efficient and clinically worthwhile for researchers to concentrate on model evaluation and updating rather than continual re-development.

### Supplementary information

Below is the link to the electronic supplementary material.

Supplementary file1 (PDF 174 kb)

## Abbreviations

IQR: Inter-quartile range
PRISMA: Preferred reporting items for systematic reviews and meta-analysis
PROBAST: Prediction model risk of bias assessment tool
TRIPOD: Transparent reporting of a multivariable prediction model for individual prognosis or diagnosis

## Declarations

### Guarantor

The scientific guarantor of this publication is SH.

### Statistics and biometry

Two of the authors have significant statistical expertise. One of the authors (SM) is a medical statistician.

### Study subjects or cohorts overlap

No study subjects or cohort overlap has been reported.

### Methodology

• Retrospective

• Systematic review

• Performed at one institution
