# Supplementary material for: Meta-research: How many diagnostic or prognostic models published in radiological journals are evaluated externally?
Source: Eur Radiol. 2023 Sep 12;34(4):2524–33. doi: 10.1007/s00330-023-10168-3 (PMC10957714; doi:10.1007/s00330-023-10168-3)
Supplement: Supplementary file 1 — Supplementary file1 (PDF 174 kb) [file 330_2023_10168_MOESM1_ESM.pdf]

# ELECTRONIC SUPPLEMENTAL MATERIAL.

List of papers selected for the systematic review:


1. Aertsen M, De Keyzer F, Van Poppel H, Joniau S, De Wever L, Lerut E, et al. Tumour-related imaging parameters predicting the percentage of preserved normal renal parenchyma following nephron sparing surgery: a retrospective study. European radiology. 2013;23(1):280-6.

2. Ahn SS, Nam HS, Heo JH, Kim YD, Lee SK, Han KH, et al. Ischemic stroke: measurement of intracranial artery calcifications can improve prediction of asymptomatic coronary artery disease. Radiology. 2013;268(3):842-9.

3. Ahn SY, Park CM, Park SJ, Kim HJ, Song C, Lee SM, et al. Prognostic value of computed tomography texture features in non-small cell lung cancers treated with definitive concomitant chemoradiotherapy. Investigative radiology. 2015;50(10):719-25.

4. An C, Kim DW, Park YN, Chung YE, Rhee H, Kim MJ. Single Hepatocellular Carcinoma: Preoperative MR Imaging to Predict Early Recurrence after Curative Resection. Radiology. 2015;276(2):433-43.

5. An C, Lee HJ, Lee HS, Ahn SS, Choi BW, Kim MJ, et al. CT-based abdominal aortic calcification score as a surrogate marker for predicting the presence of asymptomatic coronary artery disease. European radiology. 2014;24(10):2491-8.

6. Aoki T, Oshige T, Matsuyama A, Oki H, Kinoshita S, Yamashita Y, et al. High-resolution MRI predicts steroid injection response in carpal tunnel syndrome patients. European radiology. 2014;24(3):559-65.

7. Aviram G, Shmueli H, Adam SZ, Bendet A, Ziv-Baran T, Steinvil A, et al. Pulmonary Hypertension: A Nomogram Based on CT Pulmonary Angiographic Data for Prediction in Patients without Pulmonary Embolism. Radiology. 2015;277(1):236-46.

8. Bamberg F, Parhofer KG, Lochner E, Marcus RP, Theisen D, Findeisen HM, et al. Diabetes mellitus: long-term prognostic value of whole-body MR imaging for the occurrence of cardiac and cerebrovascular events. Radiology. 2013;269(3):730-7.

9. Bhosale P, Shah A, Wei W, Varadhachary G, Johnson V, Shah V, et al. Carcinoid tumours: predicting the location of the primary neoplasm based on the sites of metastases. European radiology. 2013;23(2):400-7.

10. Bodanapally UK, Van der Byl G, Shanmuganathan K, Katzman L, Geraymovych E, Saksobhavivat N, et al. Traumatic optic neuropathy prediction after blunt facial trauma: derivation of a risk score based on facial CT findings at admission. Radiology. 2014;272(3):824-31.

11. Buckens CF, van der Graaf Y, Verkooijen HM, Mali WP, Isgum I, Mol CP, et al. Osteoporosis markers on low-dose lung cancer screening chest computed tomography scans predict all-cause mortality. European radiology. 2015;25(1):132-9.

12. Cannie MM, Cordier AG, De Laveaucoupet J, Franchi-Abella S, Cagneaux M, Prodhomme O, et al. Liver-to-thoracic volume ratio: use at MR imaging to predict postnatal survival in fetuses with

isolated congenital diaphragmatic hernia with or without prenatal tracheal occlusion. European radiology. 2013;23(5):1299-305.

13. Chapiro J, Duran R, Lin M, Schernthaner R, Lesage D, Wang Z, et al. Early survival prediction after intra-arterial therapies: a 3D quantitative MRI assessment of tumour response after TACE or radioembolization of colorectal cancer metastases to the liver. European radiology. 2015;25(7):1993-2003.

14. Cook GJ, O'Brien ME, Siddique M, Chicklore S, Loi HY, Sharma B, et al. Non-Small Cell Lung Cancer Treated with Erlotinib: Heterogeneity of (18)F-FDG Uptake at PET-Association with Treatment Response and Prognosis. Radiology. 2015;276(3):883-93.

15. Diaz-Zamudio M, Dey D, Schuhbaeck A, Nakazato R, Gransar H, Slomka PJ, et al. Automated Quantitative Plaque Burden from Coronary CT Angiography Noninvasively Predicts Hemodynamic Significance by using Fractional Flow Reserve in Intermediate Coronary Lesions. Radiology. 2015;276(2):408-15.

16. Dikaios N, Alkalbani J, Sidhu HS, Fujiwara T, Abd-Alazeez M, Kirkham A, et al. Logistic regression model for diagnosis of transition zone prostate cancer on multi-parametric MRI. European radiology. 2015;25(2):523-32.

17. Domachevsky L, Groshar D, Galili R, Saute M, Bernstine H. Survival Prognostic Value of Morphological and Metabolic variables in Patients with Stage I and II Non-Small Cell Lung Cancer. European radiology. 2015;25(11):3361-7.

18. Eilaghi A, Brooks J, d'Esterre C, Zhang L, Swartz RH, Lee TY, et al. Reperfusion is a stronger predictor of good clinical outcome than recanalization in ischemic stroke. Radiology. 2013;269(1):240-8.

19. Ellingson BM, Kim HJ, Woodworth DC, Pope WB, Cloughesy JN, Harris RJ, et al. Recurrent glioblastoma treated with bevacizumab: contrast-enhanced T1-weighted subtraction maps improve tumor delineation and aid prediction of survival in a multicenter clinical trial. Radiology. 2014;271(1):200-10.

20. Emblem KE, Pinho MC, Zollner FG, Due-Tonnessen P, Hald JK, Schad LR, et al. A generic support vector machine model for preoperative glioma survival associations. Radiology. 2015;275(1):228-34.

21. Faget C, Taourel P, Charbit J, Ruyer A, Alili C, Molinari N, et al. Value of CT to predict surgically important bowel and/or mesenteric injury in blunt trauma: performance of a preliminary scoring system. European radiology. 2015;25(12):3620-8.

22. Fendler WP, Ilhan H, Paprottka PM, Jakobs TF, Heinemann V, Bartenstein P, et al. Nomogram including pretherapeutic parameters for prediction of survival after SIRT of hepatic metastases from colorectal cancer. European radiology. 2015;25(9):2693-700.

23. Giganti F, Orsenigo E, Esposito A, Chiari D, Salerno A, Ambrosi A, et al. Prognostic Role of Diffusion-weighted MR Imaging for Resectable Gastric Cancer. Radiology. 2015;276(2):444-52.

24. Gitsioudis G, Schussler A, Nagy E, Maurovich-Horvat P, Buss SJ, Voss A, et al. Combined Assessment of High-Sensitivity Troponin T and Noninvasive Coronary Plaque Composition for the Prediction of Cardiac Outcomes. Radiology. 2015;276(1):73-81.

25. Gondo T, Hricak H, Sala E, Zheng J, Moskowitz CS, Bernstein M, et al. Multiparametric 3T MRI for the prediction of pathological downgrading after radical prostatectomy in patients with biopsy-proven Gleason score 3 + 4 prostate cancer. European radiology. 2014;24(12):3161-70.

26. Gray MR, Martin del Campo S, Zhang X, Zhang H, Souza FF, Carson WE, 3rd, et al. Metastatic melanoma: lactate dehydrogenase levels and CT imaging findings of tumor devascularization allow accurate prediction of survival in patients treated with bevacizumab. Radiology. 2014;270(2):425-34.

27. Guntner O, Zeman F, Wohlgemuth WA, Heiss P, Jung EM, Wiggermann P, et al. Inferior mesenteric arterial type II endoleaks after endovascular repair of abdominal aortic aneurysm: are they predictable? Radiology. 2014;270(3):910-9.

28. Gutman DA, Cooper LA, Hwang SN, Holder CA, Gao J, Aurora TD, et al. MR imaging predictors of molecular profile and survival: multi-institutional study of the TCGA glioblastoma data set. Radiology. 2013;267(2):560-9.

29. Higaki A, Ito K, Tamada T, Sone T, Kanki A, Noda Y, et al. Prognosis of small hepatocellular nodules detected only at the hepatobiliary phase of Gd-EOB-DTPA-enhanced MR imaging as hypointensity in cirrhosis or chronic hepatitis. European radiology. 2014;24(10):2476-81.

30. Hocquelet A, Cornelis F, Le Bras Y, Meyer M, Tricaud E, Lasserre AS, et al. Long-term results of preventive embolization of renal angiomyolipomas: evaluation of predictive factors of volume decrease. European radiology. 2014;24(8):1785-93.

31. Hunter GJ, Ginat DT, Kelly HR, Halpern EF, Hamberg LM. Discriminating parathyroid adenoma from local mimics by using inherent tissue attenuation and vascular information obtained with four-dimensional CT: formulation of a multinomial logistic regression model. Radiology. 2014;270(1):168-75.

32. Hwang EJ, Lee JM, Yoon JH, Kim JH, Han JK, Choi BI, et al. Intravoxel incoherent motion diffusion-weighted imaging of pancreatic neuroendocrine tumors: prediction of the histologic grade using pure diffusion coefficient and tumor size. Investigative radiology. 2014;49(6):396-402.

33. Hwang EJ, Park CM, Ryu Y, Lee SM, Kim YT, Kim YW, et al. Pulmonary adenocarcinomas appearing as part-solid ground-glass nodules: is measuring solid component size a better prognostic indicator? European radiology. 2015;25(2):558-67.

34. Jabehdar Maralani P, Melhem ER, Wang S, Herskovits EH, Voluck MR, Kim SJ, et al. Association of dynamic susceptibility contrast enhanced MR Perfusion parameters with prognosis in elderly patients with glioblastomas. European radiology. 2015;25(9):2738-44.

35. Jain R, Poisson L, Narang J, Gutman D, Scarpace L, Hwang SN, et al. Genomic mapping and survival prediction in glioblastoma: molecular subclassification strengthened by hemodynamic imaging biomarkers. Radiology. 2013;267(1):212-20.

36. Jain R, Poisson LM, Gutman D, Scarpace L, Hwang SN, Holder CA, et al. Outcome prediction in patients with glioblastoma by using imaging, clinical, and genomic biomarkers: focus on the nonenhancing component of the tumor. Radiology. 2014;272(2):484-93.

37. Jamshidi N, Jonasch E, Zapala M, Korn RL, Aganovic L, Zhao H, et al. The Radiogenomic Risk Score: Construction of a Prognostic Quantitative, Noninvasive Image-based Molecular Assay for Renal Cell Carcinoma. Radiology. 2015;277(1):114-23.

38. Jin KN, Moon HJ, Sung YW, Lee Y, Wi JY. Preoperative computed tomography of the chest in lung cancer patients: the predictive value of calcified lymph nodes for the perioperative outcomes of video-assisted thoracoscopic surgery lobectomy. European radiology. 2013;23(12):3278-86.

39. Kendall GS, Melbourne A, Johnson S, Price D, Bainbridge A, Gunny R, et al. White matter NAA/Cho and Cho/Cr ratios at MR spectroscopy are predictive of motor outcome in preterm infants. Radiology. 2014;271(1):230-8.

40. Kim H, Kim JA, Son EJ, Youk JH. Quantitative assessment of shear-wave ultrasound elastography in thyroid nodules: diagnostic performance for predicting malignancy. European radiology. 2013;23(9):2532-7.

41. Kim JY, Lee SH, Kim S, Kang T, Bae YT. Tumour 18 F-FDG Uptake on preoperative PET/CT may predict axillary lymph node metastasis in ER-positive/HER2-negative and HER2-positive breast cancer subtypes. European radiology. 2015;25(4):1172-81.

42. Kim TH, Jeong JY, Lee SW, Kim CK, Park BK, Sung HH, et al. Diffusion-weighted magnetic resonance imaging for prediction of insignificant prostate cancer in potential candidates for active surveillance. European radiology. 2015;25(6):1786-92.

43. Kim TH, Kim JH, Shin CI, Kim SH, Han JK, Choi BI. CT findings suggesting anastomotic leak and predicting the recovery period following gastric surgery. European radiology. 2015;25(7):1958-66.

44. King AD, Chow KK, Yu KH, Mo FK, Yeung DK, Yuan J, et al. Head and neck squamous cell carcinoma: diagnostic performance of diffusion-weighted MR imaging for the prediction of treatment response. Radiology. 2013;266(2):531-8.

45. King KS, Chen KX, Hulsey KM, McColl RW, Weiner MF, Nakonezny PA, et al. White matter hyperintensities: use of aortic arch pulse wave velocity to predict volume independent of other cardiovascular risk factors. Radiology. 2013;267(3):709-17.

46. Kirby M, Pike D, Coxson HO, McCormack DG, Parraga G. Hyperpolarized (3)He ventilation defects used to predict pulmonary exacerbations in mild to moderate chronic obstructive pulmonary disease. Radiology. 2014;273(3):887-96.

47. Ko BS, Wong DT, Cameron JD, Leong DP, Leung M, Meredith IT, et al. 320-row CT coronary angiography predicts freedom from revascularisation and acts as a gatekeeper to defer invasive angiography in stable coronary artery disease: a fractional flow reserve-correlated study. European radiology. 2014;24(3):738-47.

48. Ko BS, Wong DT, Cameron JD, Leong DP, Soh S, Nerlekar N, et al. The ASLA Score: A CT Angiographic Index to Predict Functionally Significant Coronary Stenoses in Lesions with Intermediate Severity-Diagnostic Accuracy. Radiology. 2015;276(1):91-101.

49. Koo HR, Park JS, Kang KW, Han W, Park IA, Moon WK. Correlation between (18)F-FDG uptake on PET/CT and prognostic factors in triple-negative breast cancer. European radiology. 2015;25(11):3314-21.

50. Lan M, Huang Y, Chen CY, Han F, Wu SX, Tian L, et al. Prognostic Value of Cervical Nodal Necrosis in Nasopharyngeal Carcinoma: Analysis of 1800 Patients with Positive Cervical Nodal Metastasis at MR Imaging. Radiology. 2015;276(2):536-44.

51. Lassau N, Bonastre J, Kind M, Vilgrain V, Lacroix J, Cuinet M, et al. Validation of dynamic contrast-enhanced ultrasound in predicting outcomes of antiangiogenic therapy for solid tumors:

the French multicenter support for innovative and expensive techniques study. Investigative radiology. 2014;49(12):794-800.

52. Lee DH, Lee JM, Lee JY, Kim SH, Yoon JH, Kim YJ, et al. Radiofrequency ablation of hepatocellular carcinoma as first-line treatment: long-term results and prognostic factors in 162 patients with cirrhosis. Radiology. 2014;270(3):900-9.

53. Lee K, Hur J, Hong SR, Suh YJ, Im DJ, Kim YJ, et al. Predictors of Recurrent Stroke in Patients with Ischemic Stroke: Comparison Study between Transesophageal Echocardiography and Cardiac CT. Radiology. 2015;276(2):381-9.

54. Leipsic J, Taylor CM, Gransar H, Shaw LJ, Ahmadi A, Thompson A, et al. Sex-based prognostic implications of nonobstructive coronary artery disease: results from the international multicenter CONFIRM study. Radiology. 2014;273(2):393-400.

55. Lewin M, Gelu-Simeon M, Ostos M, Boufassa F, Sobesky R, Teicher E, et al. Imaging Features and Prognosis of Hepatocellular Carcinoma in Patients with Cirrhosis Who Are Coinfected with Human Immunodeficiency Virus and Hepatitis C Virus. Radiology. 2015;277(2):443-53.

56. Li M, Zhang J, Pan J, Lu Z. Coronary total occlusion lesions: linear intrathrombus enhancement at CT predicts better outcome of percutaneous coronary intervention. Radiology. 2013;266(2):443-51.

57. Loffroy R, Lin M, Yenokyan G, Rao PP, Bhagat N, Noordhoek N, et al. Intraprocedural C-arm dual-phase cone-beam CT: can it be used to predict short-term response to TACE with drug-eluting beads in patients with hepatocellular carcinoma? Radiology. 2013;266(2):636-48.

58. Masarwah A, Auvinen P, Sudah M, Rautiainen S, Sutela A, Pelkonen O, et al. Very low mammographic breast density predicts poorer outcome in patients with invasive breast cancer. European radiology. 2015;25(7):1875-82.

59. Meyrignac O, Lagarde S, Bournet B, Mokrane FZ, Buscail L, Rousseau H, et al. Acute Pancreatitis: Extrapancreatic Necrosis Volume as Early Predictor of Severity. Radiology. 2015;276(1):119-28.

60. Millet I, Curros-Doyon F, Molinari N, Bouic-Pages E, Prat X, Alili C, et al. Invasive breast carcinoma: influence of prognosis and patient-related factors on kinetic MR imaging characteristics. Radiology. 2014;270(1):57-66.

61. Morsbach F, Pfammatter T, Reiner CS, Fischer MA, Sah BR, Winklhofer S, et al. Computed tomographic perfusion imaging for the prediction of response and survival to transarterial radioembolization of liver metastases. Investigative radiology. 2013;48(11):787-94.

62. Morsbach F, Sah BR, Spring L, Puippe G, Gordic S, Seifert B, et al. Perfusion CT best predicts outcome after radioembolization of liver metastases: a comparison of radionuclide and CT imaging techniques. European radiology. 2014;24(7):1455-65.

63. Muller-Wille R, Schotz S, Zeman F, Uller W, Guntner O, Pfister K, et al. CT features of early type II endoleaks after endovascular repair of abdominal aortic aneurysms help predict aneurysm sac enlargement. Radiology. 2015;274(3):906-16.

64. Noda Y, Kanematsu M, Goshima S, Suzui N, Hirose Y, Matsunaga K, et al. 18-F fluorodeoxyglucose uptake in positron emission tomography as a pathological grade predictor for renal clear cell carcinomas. European radiology. 2015;25(10):3009-16.

65. Park JJ, Chun EJ, Cho YS, Oh IY, Yoon CH, Suh JW, et al. Potential predictors of side-branch occlusion in bifurcation lesions after percutaneous coronary intervention: a coronary CT angiography study. Radiology. 2014;271(3):711-20.

66. Park JJ, Kim CK, Park SY, Park BK, Kim B. Value of diffusion-weighted imaging in predicting parametrial invasion in stage IA2-IIA cervical cancer. European radiology. 2014;24(5):1081-8.

67. Pickles MD, Lowry M, Manton DJ, Turnbull LW. Prognostic value of DCE-MRI in breast cancer patients undergoing neoadjuvant chemotherapy: a comparison with traditional survival indicators. European radiology. 2015;25(4):1097-106.

68. Ravanelli M, Farina D, Morassi M, Roca E, Cavalleri G, Tassi G, et al. Texture analysis of advanced non-small cell lung cancer (NSCLC) on contrast-enhanced computed tomography: prediction of the response to the first-line chemotherapy. European radiology. 2013;23(12):3450-5.

69. Riches SF, Payne GS, Morgan VA, Dearnaley D, Morgan S, Partridge M, et al. Multivariate modelling of prostate cancer combining magnetic resonance derived T2, diffusion, dynamic contrast-enhanced and spectroscopic parameters. European radiology. 2015;25(5):1247-56.

70. Rosenkrantz AB, Sekhar A, Genega EM, Melamed J, Babb JS, Patel AD, et al. Prognostic implications of the magnetic resonance imaging appearance in papillary renal cell carcinoma. European radiology. 2013;23(2):579-87.

71. Satoh Y, Onishi H, Nambu A, Araki T. Volume-based parameters measured by using FDG PET/CT in patients with stage I NSCLC treated with stereotactic body radiation therapy: prognostic value. Radiology. 2014;270(1):275-81.

72. Schmit GD, Schenck LA, Thompson RH, Boorjian SA, Kurup AN, Weisbrod AJ, et al. Predicting renal cryoablation complications: new risk score based on tumor size and location and patient history. Radiology. 2014;272(3):903-10.

73. Shaffer JL, Petrella JR, Sheldon FC, Choudhury KR, Calhoun VD, Coleman RE, et al. Predicting cognitive decline in subjects at risk for Alzheimer disease by using combined cerebrospinal fluid, MR imaging, and PET biomarkers. Radiology. 2013;266(2):583-91.

74. Sinha A, Singh VK, Cruise M, Afghani E, Matsukuma K, Ali S, et al. Abdominal CT predictors of fibrosis in patients with chronic pancreatitis undergoing surgery. European radiology. 2015;25(5):1339-46.

75. Sohn B, Lim JS, Kim H, Myoung S, Choi J, Kim NK, et al. MRI-detected extramural vascular invasion is an independent prognostic factor for synchronous metastasis in patients with rectal cancer. European radiology. 2015;25(5):1347-55.

76. Sommer WH, Ceelen F, Garcia-Albeniz X, Paprottka PM, Auernhammer CJ, Armbruster M, et al. Defining predictors for long progression-free survival after radioembolisation of hepatic metastases of neuroendocrine origin. European radiology. 2013;23(11):3094-103.

77. Soussan M, Cyrta J, Pouliquen C, Chouahnia K, Orlhac F, Martinod E, et al. Fluorine 18 fluorodeoxyglucose PET/CT volume-based indices in locally advanced non-small cell lung cancer: prediction of residual viable tumor after induction chemotherapy. Radiology. 2014;272(3):875-84.

78. Ulaner GA, Goldman DA, Sauter CS, Migliacci J, Lilienstein J, Gonen M, et al. Prognostic Value of FDG PET/CT before Allogeneic and Autologous Stem Cell Transplantation for Aggressive Lymphoma. Radiology. 2015;277(2):518-26.

79. Vandecaveye V, Michielsen K, De Keyzer F, Laleman W, Komuta M, Op de beeck K, et al. Chemoembolization for hepatocellular carcinoma: 1-month response determined with apparent diffusion coefficient is an independent predictor of outcome. Radiology. 2014;270(3):747-57.

80. Vargas HA, Micco M, Hong SI, Goldman DA, Dao F, Weigelt B, et al. Association between morphologic CT imaging traits and prognostically relevant gene signatures in women with high-grade serous ovarian cancer: a hypothesis-generating study. Radiology. 2015;274(3):742-51.

81. Vogl TJ, Dommermuth A, Heinle B, Nour-Eldin NE, Lehnert T, Eichler K, et al. Colorectal cancer liver metastases: long-term survival and progression-free survival after thermal ablation using magnetic resonance-guided laser-induced interstitial thermotherapy in 594 patients: analysis of prognostic factors. Investigative radiology. 2014;49(1):48-56.

82. Vogl TJ, Freier V, Nour-Eldin NE, Eichler K, Zangos S, Naguib NN. Magnetic resonance-guided laser-induced interstitial thermotherapy of breast cancer liver metastases and other noncolorectal cancer liver metastases: an analysis of prognostic factors for long-term survival and progression-free survival. Investigative radiology. 2013;48(6):406-12.

83. Wang D, Gaba RC, Jin B, Lewandowski RJ, Riaz A, Memon K, et al. Perfusion reduction at transcatheter intraarterial perfusion MR imaging: a promising intraprocedural biomarker to predict transplant-free survival during chemoembolization of hepatocellular carcinoma. Radiology. 2014;272(2):587-97.

84. Wang X, You JJ. Head CT for nontrauma patients in the emergency department: clinical predictors of abnormal findings. Radiology. 2013;266(3):783-90.

85. Wen CY, Cui JL, Liu HS, Mak KC, Cheung WY, Luk KD, et al. Is diffusion anisotropy a biomarker for disease severity and surgical prognosis of cervical spondylotic myelopathy? Radiology. 2014;270(1):197-204.

86. Wilczek ML, Kalvesten J, Algulin J, Beiki O, Brismar TB. Digital X-ray radiogrammetry of hand or wrist radiographs can predict hip fracture risk--a study in 5,420 women and 2,837 men. European radiology. 2013;23(5):1383-91.

87. Wu YC, Hsu HH, Chang WC, Tung HJ, Ko KH, Hsu YC, et al. Prognostic potential of initial CT changes for progression-free survival in gefitinib-treated patients with advanced adenocarcinoma of the lung: a preliminary analysis. European radiology. 2015;25(6):1801-13.

88. Yanagawa M, Tanaka Y, Leung AN, Morii E, Kusumoto M, Watanabe S, et al. Prognostic importance of volumetric measurements in stage I lung adenocarcinoma. Radiology. 2014;272(2):557-67.

89. Yip C, Landau D, Kozarski R, Ganeshan B, Thomas R, Michaelidou A, et al. Primary esophageal cancer: heterogeneity as potential prognostic biomarker in patients treated with definitive chemotherapy and radiation therapy. Radiology. 2014;270(1):141-8.

90. Yoon HJ, Kim Y, Kim BS. Intratumoral metabolic heterogeneity predicts invasive components in breast ductal carcinoma in situ. European radiology. 2015;25(12):3648-58.

91. Yoon YE, Kitagawa K, Kato S, Nakajima H, Kurita T, Dohi K, et al. Prognostic value of unrecognised myocardial infarction detected by late gadolinium-enhanced MRI in diabetic patients with normal global and regional left ventricular systolic function. European radiology. 2013;23(8):2101-8.

92. Yoshida S, Kobayashi S, Koga F, Ishioka J, Ishii C, Tanaka H, et al. Apparent diffusion coefficient as a prognostic biomarker of upper urinary tract cancer: a preliminary report. European radiology. 2013;23(8):2206-14.

93. Zeng L, Huang SM, Tian YM, Sun XM, Han F, Lu TX, et al. Normal Tissue Complication Probability Model for Radiation-induced Temporal Lobe Injury after Intensity-modulated Radiation Therapy for Nasopharyngeal Carcinoma. Radiology. 2015;276(1):243-9.

94. Zhang GY, Huang Y, Cai XY, Chen XP, Xu T, Wu J, et al. Prognostic value of grading masticator space involvement in nasopharyngeal carcinoma according to MR imaging findings. Radiology. 2014;273(1):136-43.

95. Zhang H, Graham CM, Elci O, Griswold ME, Zhang X, Khan MA, et al. Locally advanced squamous cell carcinoma of the head and neck: CT texture and histogram analysis allow independent prediction of overall survival in patients treated with induction chemotherapy. Radiology. 2013;269(3):801-9.

96. Zhao F, Ahlawat S, Farahani SJ, Weber KL, Montgomery EA, Carrino JA, et al. Can MR imaging be used to predict tumor grade in soft-tissue sarcoma? Radiology. 2014;272(1):192-201.

97. Zhou C, Duan X, Lan B, Liao J, Shen J. Prognostic CT and MR imaging features in patients with untreated extranodal non-Hodgkin lymphoma of the head and neck region. European radiology. 2015;25(10):3035-42.

98. Zhu Y, Deng YB, Liu YN, Bi XJ, Sun J, Tang QY, et al. Use of carotid plaque neovascularization at contrast-enhanced US to predict coronary events in patients with coronary artery disease. Radiology. 2013;268(1):54-60.
